# Sulfadiazine analogs: anti*-Toxoplasma* in vitro study of *s*ulfonamide triazoles

**DOI:** 10.1007/s00436-023-07936-x

**Published:** 2023-08-23

**Authors:** Fadwa M Arafa, Doaa Hassan Osman, Mona Mohamed Tolba, Nadjet Rezki, Mohamed R Aouad, Mohamed Hagar, Mervat Osman, Heba Said

**Affiliations:** 1 Department of Medical Parasitology, Faculty of Medicine, Alexandria University, Alexandria, 21577 Egypt; 2 Department of Parasitology, Medical Research Institute, Alexandria University, Alexandria, 21561 Egypt; 3 Department of Chemistry, College of Science, Taibah University, Al-Madinah Al-Munawarah, 30002 Saudi Arabia; 4 Department of Chemistry, Faculty of Science, Alexandria University, Alexandria, 21321 Egypt

**Keywords:** *Toxoplasma gondii*, In vitro study, 1,2,3-triazoles, Click chemistry, SEM, TEM

## Abstract

Toxoplasmosis is an infection that prevails all over the world and is caused by the obligate intracellular protozoan parasite *Toxoplasma gondii (T. gondii).* Promising novel compounds for the treatment of *T. gondii* are introduced in the current investigation. In order to test their in vitro potency against *T. gondii* tachyzoites, six 1,2,3-triazoles-based sulfonamide scaffolds with terminal NH_2_ or OH group were prepared and investigated as sulfadiazine equivalents. When compared to sulfadiazine, which served as a positive control, hybrid molecules showed much more anti-*Toxoplasma* activity. The results showed that the IC_50_ of the examined compounds **3**(**a**–**f**) were recoded as **0.07492 μM, 0.07455 μM, 0.0392 μM, 0.03124 μM**, **0.0533 μM**, and **0.01835 μM,** respectively, while the sulfadiazine exhibited **0.1852 μM**. The studied 1,2,3-triazole-sulfadrug molecular conjugates **3**(**a**–**f**) revealed selectivity index of **10.4, 8.9, 25.4, 21, 8.3, and 29**; respectively. The current study focused on the newly synthesized amino derivatives **3**(**d**–**f**), as they contain the more potent amino groups which are recognized to be essential elements and promote better biological activity. Extracellular tachyzoites underwent striking morphological alterations after 2 h of treatment as seen by scanning electron microscopy (SEM). Additionally, the intracellular tachyzoite exposed to the newly synthesized amino derivatives **3**(**d**–**f**) for a 24-h period of treatment revealed damaged and altered morphology by transmission electron microscopic (TEM) indicating cytopathic effects. Moreover, compound **3f** underwent the most pronounced changes, indicating that it had the strongest activity against *T. gondii.*

**Supplementary Information:**

The online version contains supplementary material available at 10.1007/s00436-023-07936-x.

## Introduction

Almost all warm-blooded animals are susceptible to the opportunistic, zoonotic, and obligate intracellular coccidian protozoan known as *Toxoplasma gondii* (*T. gondii*) (Dubey 2016). Up to one-third of people worldwide are infected with *T. gondii* according to the World Health Organization (WHO) (Hermes et al. 2008). Tachyzoites, tissue cysts with bradyzoites, and mature oocysts with sporozoites are the three main infective stages of *T. gondii* (Ozgonul and Besirli 2017). Despite having a single species, *Toxoplasma* possesses several clonal lineages that differ in their pathogenicity (Sanchez and Besteiro 2021), of which, type I (RH strain in the present study) has the highest virulence and is lethal at all doses in all strains of mice during the acute stage of the disease (Boyle et al. 2006). Moreover, it is already known that the parasite is highly diverse in South America, and also North America shows circulating atypical populations (Galal et al. 2019).

Toxoplasmosis is the disease caused by this parasite, and it can affect humans in both acute and chronic forms (Al-Malki 2021). The rapidly proliferative tachyzoites enter the cells during the acute stage, where cell penetration necessitates attachment of the anterior tip of the tachyzoite to the host cell (Wong and Remington 1993). They can turn into bradyzoites with the formation of tissue cysts in chronic stage (Paredes-Santos et al. 2013). The release of bradyzoites occurs when these tissue cysts rupture especially in immunocompromised individuals. Then, the disease is reactivated as a result of their conversion to tachyzoites. The pathogenesis and protracted nature of infection are both dependent on the tachyzoite-bradyzoite conversion pathway (Howe and Sibley 1995). During the acute phase of toxoplasmosis, the rapidly multiplying tachyzoites are responsible for the numerous necrotic changes and destruction of the host cells causing retinochoroiditis and meningoencephalitis in immunocompromised patients (Choi et al. 1997; Park et al. 2011). Additionally, *T. gondii* is often linked to abortion and congenital infection (Thebault et al. 2021).

**Fig. 1:**
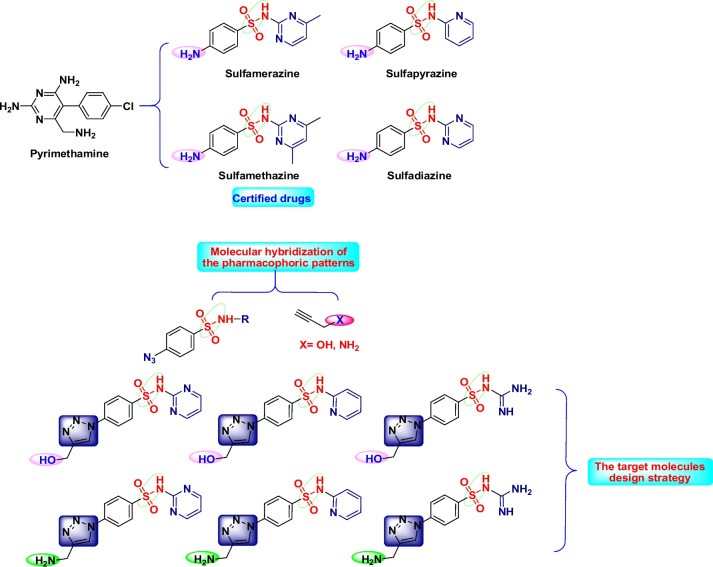
Rational design of the target molecules with alignment to the approved medications

Inhibiting the folate pathway of the parasite is currently the primary line of treatment for toxoplasmosis (Anderson 2005; Wei et al. 2015). This can be achieved using sulfa drugs, also known as sulfonamides, which are well known as the oldest synthesized antimicrobial agents with distinct properties that make them a promising candidate in the treatment and prevention of infections in humans (Tacic et al, 2017). The most efficient certified drugs are pyrimethamine-derived sulfadiazine, sulfamerazine, sulfamethazine, and sulfapyridine (Fig. 1) as the pyrimethamine is constantly present in the greater part of drug treatments. Combinations of sulfonamides and 2,4-diaminopyrimidines, such as sulfadiazine and pyrimethamine, are the most often used treatments (Saraf et al. 2017). The sulfonamide component inhibits dihydropteroate synthase, a crucial enzyme used by the parasite that produces 4-aminobenzoic acid in the critical biosynthesis of dihydropteroic acid. While dihydrofolate reductase, an enzyme required for converting dihydropteroic acid to tetrahydrofolate, is blocked by the 2,4-diaminopyrimidine component, making these combinations highly synergistic. Together, these elements prevent the growth of the parasite by preventing the manufacturing of tetrahydrofolate, a vital component needed for the creation of nucleic acids, which are necessary for DNA synthesis (Wei et al. 2015). However, pyrimethamine is linked to serious side effects, such as anemia brought on by bone marrow suppression that need folic acid co-administration (leucovorin). Moreover, sulfadiazine causes anaphylactic reactions, hypersensitivity, and acute renal failure mainly because of high medication dose, which sometimes requires treatment discontinuation (Kongsaengdao et al. 2008; McGettigan et al. 2012). There is a critical need for novel drugs or drug combinations with higher therapeutic efficacy because till now there has not yet been discovered a way to entirely eradicate the parasite from an infected organism (Pink et al. 2005).

Recently, the molecular hybridization approach became a revolutionary strategy in drug design which involves combining pharmacophoric conjugates of distinct bioactive molecules to create a new hybrid framework called “hybrid-molecule” with enhanced affinity to its target receptor and efficacy over the parent drugs (Bérubé 2016; Molina et al. 2021; Viegas-Junior et al. 2007). The tunable 1,2,3-triazole scaffolds are being the horizon for many researchers (Kumar et al. 2021; Sahu et al. 2020), and their demanding role in drug discovery and synthesis has been steadily undertaken due to their fascinating pharmacological properties (Celik et al. 2018), especially the antiparasitic activity. Some of its conjugates have been certified for use in clinics and hospitals; others are in clinical trials to treat a variety of parasites (Hernandez et al. 2017). Sulfa drugs are well defined as the basis of a quiet revolution in medicinal chemistry (Jeliński et al. 2019; Smith and Powell 2000).

In light of these findings on the promising bio-activities of sulfa drugs and 1,4-disubstituted-1,2,3-triazole derivatives, these intriguing scaffolds motivated us to design and generate new focused 1,2,3-triazole-sulfonamide molecular conjugates that simulate the perfectly matched inhibition properties of *T. gondii* of the certified drugs as continuation to our previous work (Aljohani et al. 2022). In the present work, we focused our design on mimicking the certified medications, notably the most potent amino groups, which are known to be critical attributes in the interaction with the receptor protein via hydrogen bonding, resulting in enhanced biological activity (Craik et al. 2013). We report herein the design and synthesis of sulfonamide-1,2,3-triazole molecular hybrids using click chemistry protocol and the investigation of their therapeutic efficacy against *T.gondii* tachyzoites in comparison with sulfadiazine in vitro in Vero cell line culture.

## Materials and methods

### Chemistry

All used solvents and reagents were of the greatest analytical reagent grade and were not further purified. Stuart Scientific SMP1 was used to determine the melting points and are uncorrected**.** TLC was carried out on UV fluorescent Silica gel Merck 60 F254 plates, and the spots were identified with a UV lamp (254 nm). The SHIMADZU FTIR-Affinity-1S spectrometer was used to identify the main functional groups ranging from 400 to 4000 cm^−1^. While, Bruker spectrometer (400 MHz) was used to collect the NMR spectra using tetramethyl silane (TMS) as an internal reference. The high-resolution mass spectroscopy (HRMS) was performed using the LCMS/MS impact II. GmbH-Vario EL III Elementar Analyzer was used to perform the elemental analyses.

### Synthesis of sulfonamide-based 1, 2, 3-triazoles **3**(**a**–**f**)

To a solution of propargyl amine or alcohol (1 mmol) in DMSO (10 ml) was added a solution of copper sulfate (0.10 g) and sodium ascorbate (0.15 g) in water (10 ml) drop wise under stirring. The appropriate sulfa drug azide **2a**–**c** (1 mmol) was then added to the reaction mixture; the stirring was continued for 6–10 h at room temperature. The reaction was monitored by TLC (hexane-ethyl acetate), and once it was completed, crushed ice water was added to the mixture. Filtration was used to collect the precipitate formed, which was then washed with saturated ammonium chloride solution before being recrystallized from ethanol/DMF to yield the required 1,2,3-triazoles **3**(**a**–**f**). The detailed characterization of the prepared compound is shown in Supplementary data.

### Maintenance of *Toxoplasma* strain

Virulent *T. gondii* RH strain was maintained in the Medical Parasitology Department, Faculty of Medicine, Alexandria University by serial intraperitoneal passages into Swiss albino mice. Peritoneal exudates were harvested on the fifth day post inoculation. Parasites were passed twice through a 27-gauge needle, washed twice by 1000 × *g* centrifugation for 10 min in RPMI 1640 without fetal bovine serum (FBS) (Gibco BRL). Then the parasites were suspended in the same medium to a density of 1×10^6^ parasites/ml. The viability was evaluated using a dye-exclusion test with 0.2% Trypan blue (Carvalho and De Melo 2010a; Conseil et al. 1999).

### Vero cell line

African green monkey kidney fibroblast cell line (Vero cell) was purchased from National Cancer Institute, Cairo, Egypt, and maintained in Medical Research Institute. Cells were grown in RPMI-1640 supplemented with 10% FBS (Gibco BRL) and protected with 1% penicillin/streptomycin solution.

### Cytotoxicity tests

Cytotoxicity tests for each single drug of the prepared sulfa drugs **3**(**a**–**f**) and sulfadiazine as positive control were performed through using (3-(4, 5-dimethylthiazol-2-yl) -2,5- diphenyl tetrazolium bromide) MTT assay. Vero cells were seeded at a density of 1×10^4^ cells/well in 96-well plate and incubated for 24 h at 37 °C in a 5% CO_2_ incubator. Cells were treated with each drug of **3**(**a**–**f**) and sulfadiazine in serial dilutions then incubated for 24 h. A stock solution in DMSO was initially prepared then diluted 100 times with culture media to obtain the highest concentration, then it was serially diluted. Three replicates for each drug concentration were performed. Cell viability was assayed by MTT method where 20 μl of 5 mg/ml MTT (Sigma, USA) was added to each well, and the plate was incubated at 37 °C for 3 h. Then, MTT solution was removed, 100 μl DMSO was added, and the absorbance of each well was measured using a Benchmark Microplate Reader (Bio Rad). Cytotoxicity was expressed as CC_50_ which was defined as the concentration of test samples that causes 50% destruction of cells (Guo et al. 2021; Montazeri et al. 2020). Experiments were repeated three times. Vero cell growth suppression (%) was estimated using the following equation:

$$\frac{\textrm{The}\ \textrm{absorbance}\ \textrm{of}\ \textrm{cells}\ \textrm{treated}\ \textrm{with}\ \textrm{sulfadrug}}{\textrm{The}\ \textrm{absorbance}\ \textrm{of}\ \textrm{cells}\ \textrm{cultured}\ \textrm{with}\ \textrm{medium}\ \textrm{alone}}\times 100$$

using CompuSyn software (version 1) (Chou 2006; Chou and Talaly 1977).

### Effects of sulfa drugs on intracellular *Toxoplasma* gondii

For this purpose, Vero cells were cultured in 96-well plates (1×10^4^ cells/well) for 24 h in RPMI 1640 medium supplemented with 10% inactivated FBS at 37 °C and 5% CO_2_. Next, the cells were infected with *T. gondii* tachyzoites (parasite: cell ratio = 10:1). Four hours following the inoculation, the cells were washed twice with RPMI to remove any non-adherent parasites. After 24 h, the medium was changed, and the infected cells were treated with serial dilutions of each drug of **3**(**a**–**f**) and sulfadiazine, and three replicates for each drug concentration were incubated for 24 h. Cell viability was assayed by MTT method. Twenty microliters of 5 mg/ml MTT (Sigma, USA) was added to each well and the plate was incubated at 37 °C for 3 h. Then, MTT solution was removed, 100 μl DMSO was added, and the absorbance of each well was measured using a Benchmark Microplate Reader (Bio Rad). The optical absorbance was measured at 570-nm wavelength. Growth inhibition (GI) was calculated as in the following equation:

$$GI\ \left(\%\right)=\left[\left( At- Ac\right)/ Ac\right]\times 100$$

where At and Ac are the absorbance of treated cells and control, respectively. In addition, IC_50_ is the 50% growth inhibition concentration. Selectivity index (SI) of the samples was calculated using the IC_50_ and the host-cell cytotoxicity profiles CC_50_ (SI = CC_50_/IC_50_) (Montazeri et al. 2020) using CompuSyn software (version 1) (Chou 2006; Chou and Talaly 1977).

### Scanning electron microscopy (SEM)

Electron microscopic analysis was performed to further explore the anti*-Toxoplasma* mechanism of the newly synthesized sulfa drugs **3**(**d**–**f**); the ultrastructure of *T. gondii* tachyzoites treated in vitro with sulfa drugs **3**(**d**–**f**) was observed using (SEM) (Joel JSM-53001A, Tokyo, Japan). Tachyzoites were collected from peritoneal exudates of infected mice on the fifth day post inoculation as previously described (Carvalho and De Melo 2010a). Then tachyzoites were divided into four tubes, each containing 1×10^5^ tachyzoites. The first tube was used as control (normal, non-treated group), while sulfa drugs **3**(**d**–**f**) were added to the remaining three tubes respectively. Then, tachyzoites were incubated for 2 h at room temperature. After that, tachyzoites were fixed with 2% paraformaldehyde and 2.5% glutaraldehyde in 0.1 M sodium cacodylate buffer (pH 7.4) washed in cacodylate buffer and attached on a slide. Then, the slide was post-fixed for 2–4 h using 1–2% osmium tetroxide in 0.1 M phosphate buffer (pH 7.2) at room temperature and dehydrated in graded ethanol dilutions (70, 80, 90, and 100%). They were dried, mounted on stubs, coated with gold (20–30 nm), and then observed using SEM (de Souza and Attias 2018; Khosravi et al. 2020).

### Transmission electron microscopy (TEM)

After confluence of Vero cells in four T-25 culture flasks, *T. gondii* RH strain tachyzoites, suspended in 5 ml of RPMI, and were added to each flasks in a ratio of 5:1 parasite-host cell (Diab and El-Bahy 2008). Flasks were incubated for 2 h. After that, the cells were washed twice with culture media to remove extracellular parasites. The cells in culture flasks were incubated in 5 ml of culture media for 24 h at 37 °C in a 5% CO_2_ (Carvalho and De Melo 2010b). The first flask was non-treated, and the other three flasks were treated with the IC_50_ of each compound (**0.3124 μM**, **0.0533 μM**, **0.01835 μM** for **3d**, **3e**, and **3f** respectively) for 24 h. For TEM (Jeol JSM-1400), trypsinization was followed by centrifugation at 2000*g* for 10 min, and the resulting pellet was fixed in buffered glutaraldehyde-phosphate 2.5% and stored at 4 °C until used (Shaw et al. 2002). Then, the fixed specimens were washed thoroughly with Millonig phosphate buffer and post-fixed with buffered osmium tetroxide-phosphate. Following that, they were dehydrated in ascending concentrations of ethyl alcohol followed by embedding in epoxy resin. Finally, ultrathin sections were doubly stained with uranyl acetate and lead citrate trihydrate stains and examined under TEM (Winey et al. 2014).

## Results

### Chemistry

**Scheme 1:**
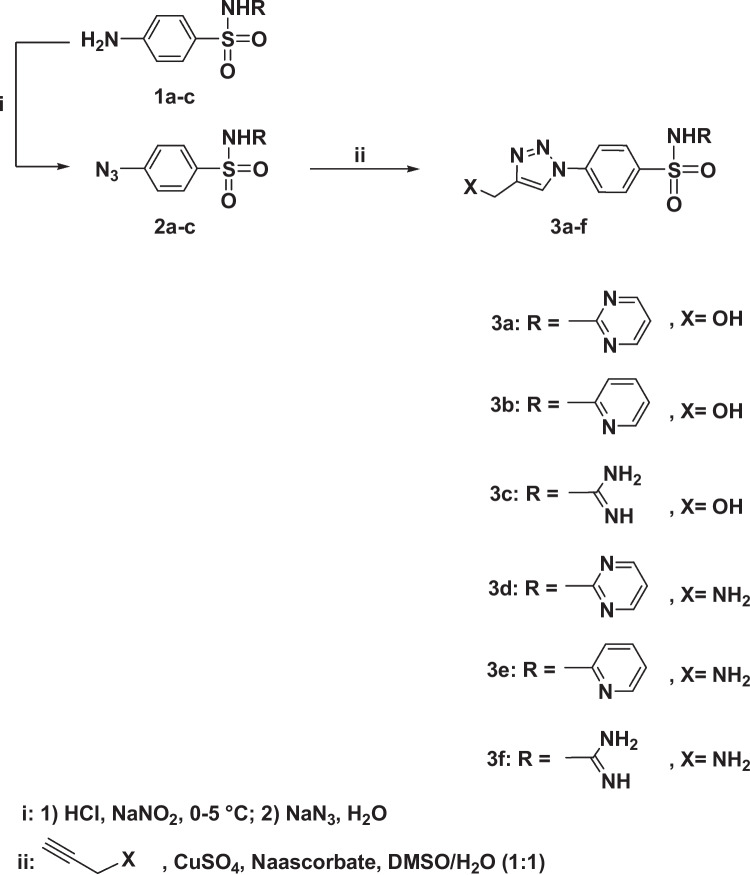
Synthesis of 1,2,3-triazole-sulfonamide hybrids **3**(**a**–**f**)

The targeted 1,2,3-triazoles-based sulfonamide was successfully synthesized through the Cu(I)-click chemistry approach (Huisgen 1963) as illustrated in Scheme 1. The click 1,3-dipolar cycloaddition reaction requires two coupling building blocks incorporating an azide side chain and a terminal alkyne. Initially, the commercially sulfa drugs **1a**–**c** undergo well-established diazotization followed by azidolysis reactions and afforded exclusively the corresponding sulfonamide azide derivatives **2a**–**c** as key intermediates (Ryu and Emrick 2011). Through the copper (I) catalyzed 1,3-dipolar cycloaddition reaction of the freshly synthesized azides **2a**–**c** and propargyl amine or alcohol were linked to yield regioselectivity the targeted 1,2,3-triazole-sulfonamide molecular hybrids **3**(**a**–**f**). The click reactions were carried out at room temperature in the presence of catalytic amount of copper sulfate and sodium ascorbate and a mixture of DMSO: water as solvent (Scheme 1).

Based on the spectral data, the structures of the resulting 1,2,3-triazole-sulfonamide molecular conjugates **3**(**a**–**f**) were deduced. Their IR spectra disclosed the absence of ≡C–H and C≡C, proving their involvement in the cycloaddition reaction. The spectra also revealed the presence of new characteristic absorption bands at 3310–3460 cm^−1^ assigned to the amino groups (OH, NH, and NH2).

The ^1^H NMR spectra of click adducts **3**(**a**–**f**) indicated clearly the disappearance of the signal attributed to the acetylenic proton (≡C–**H**) of the respected propargyl amine or alcohol and the appearance of a distinct singlet at δ_H_ 8.50–8.88 ppm, which was assigned to the **H**-5 triazolyl proton. In addition, the spectra revealed also the presence of characteristic singlets at δ_H_ 4.25–4.43, 6.78–6.85, and 12.02–12.43 ppm related to NC**H**_**2**_, N**H**_**2**_, and N**H**SO_2_ protons, respectively. The aromatic protons were recorded in the aromatic region (see Experimental section).

Moreover, their ^13f^NMR spectra also confirmed the success of the dipolar cycloaddition reaction. All spectra showed clearly the disappearance of the signals attributed to the two sp-carbons (**C**≡**C**). In addition, the signals recorded at δ_C_ 56.56–57.09 ppm were assigned to the N**C**H_2_-carbon, and the signals belonging to the aromatic carbons were observed at δ_C_ 118.87–159.75 ppm.

### Drug-likeness parameters (Lipinski rule of 5)

The drug likeness parameters were calculated using ADME calculator (Ertl et al. 2000; Lipinski et al. 2012); the results were tabulated in Table 1; it is obvious that compound **3f** is the best compared with values to the gold standerd sulfadiazine.

**Table 1:** Calculated drug-likeness parameters of **3**(**a**–**f**) and sulfadiazine

Parameter	3a	3b	3c	3d	3e	3f	Sulfadiazine	Reference value
Number of H bond donors	1	1	3	2	2	4	2	<5
Number of H bond acceptors	9	8	9	9	8	9	5	<10
LogP	−2.003	−1.304	−1.424	−1.484	−1.371	−2.070	0.86	<5
Mol. Refractivity	87.2	90.2	72.8	74.49	91.82	88.86	63.7	40–130
TPSA [Angstrom^2]	95.2	82.84	70.48	70.48	82.84	95.2	97.97	<140

### Cytotoxicity test

In the present work, the toxicity of different concentrations of the synthesized 1,2,3-triazoles-based sulfa drug core **3**(**a**–**f**) was evaluated with respect to the commercially available sulfadiazine on Vero cells using MTT test.

The results were expressed as the percent of viable cells (**CC**_**50**_) of serial diluted concentration. The results showed diverse degrees of toxicity on Vero cells. Sulfadiazine showed the highest **CC**_**50**_ (**1.3903 μM**) while tested derivatives **3a**, **3b**, **3c**, **3d**, **3e**, and **3f** showed **CC**_**50**_ *values of*^*.*^ **0.779 μM**, **0.6615 μM**, **0.9966 μM**, **0.6526 μM**, **0.5096 μM**, and **0.5405 μM** respectively.

### Effects of sulfa drugs on intracellular *Toxoplasma gondii*

Further evaluation of the ability of the investigated triazoles tethering sulfonamide linkage **3**(**a**–**f**) and the sulphadiazine to inhibit the intracellular tachyzoite proliferation within Vero cells was carried out using the MTT assay at 24-h post-treatment. The results were summarized in Table 2 where the absorbance represents the number of living Vero cells, as parasites will damage living Vero cells during proliferation and invasion; therefore, the absorbance could reflect the inhibitory effect of the tested compounds against parasites indirectly. All the evaluated compounds **3**(**a**–**f**) had more potent activity against *T. gondii* compared to that exhibited by the sulfadiazine as drug control. The derivative **3f** exhibited the highest activity against *T. gondii* with the lowest IC_50_ value of **0.01835 μM**, which is ten times lower than the highest IC_50_ value (0.**1852 μM**) recorded by the sulfadiazine (Table 2).

**Table 2:** In vitro cytotoxicity, anti-*Toxoplasma* activity, and selectivity index of the investigated compounds in comparison to sulfadiazine

Tested drug	CC_50_ (μM)	IC_50_ (μM)	SI*
3a	0.779±0.08	0.07492±0.0103	10.4
3b	0.6615±0.05	0.07455±0.003	8.9
3c	0.9966±0.06	0.0392±0.001	25.4
3d	0.6526±.0.04	0.03124±0.001	21
3e	0.5096±.022	0.0533±0.0105	8.3
3f	0.5405±.041	0.01835±0.0008	29
Sulfadiazine	1.3903±0.1	0.1852±0.0184	6.9

***CC***_***50***_, 50% cytotoxic concentration; ***C***_***50***_, inhibitory concentration; ***SI***, selectivity index

### Effects of chemical compounds on parasite invasion and in vitro replication

According to the results of the current study, the SI* of the different compounds were obtained in the following order: **3f > 3c > 3d** > **3a > 3b** > **3e > sulfadiazine**. Thus, all the synthesized sulfa drugs, especially **3f**, showed an anti-*T. gondii* activity higher than that of the positive control drug. The three newly synthesized hybrid **3**(**d**–**f**) were selected for additional investigations to illustrate their effects and mechanism of action by SEM and TEM.

### Scanning electron microscopy analysis

**Fig. 2:**
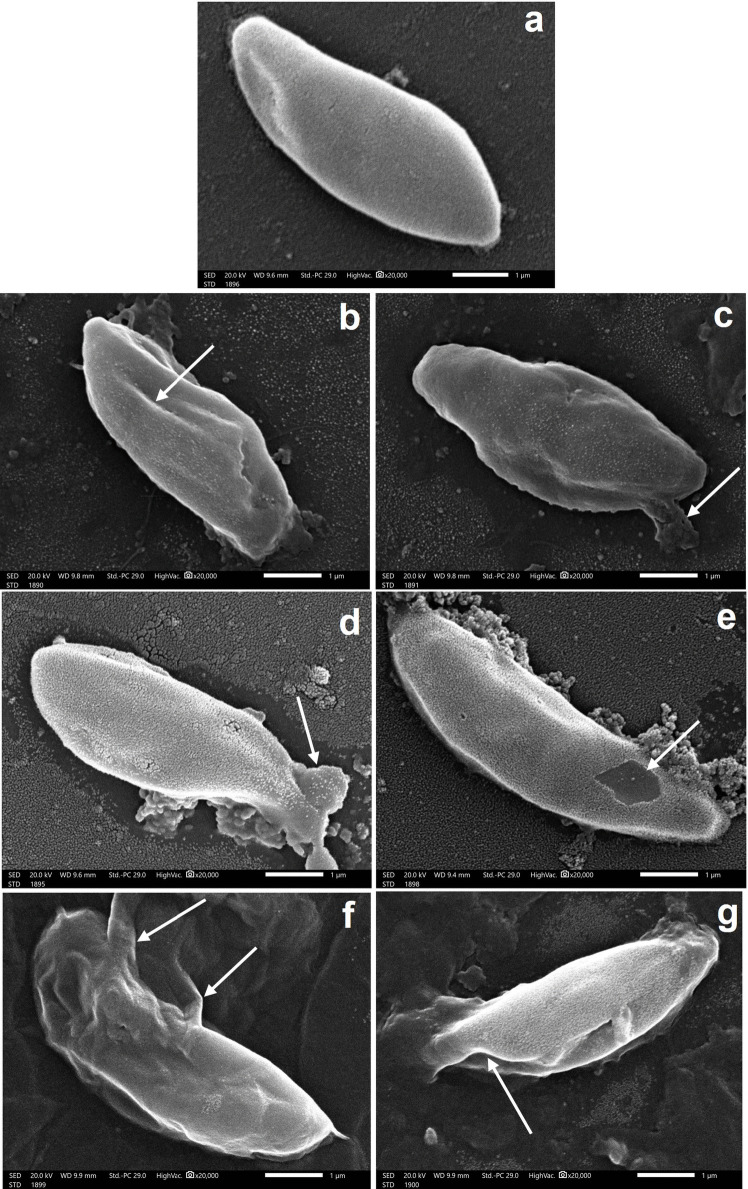
Scanning electron microscopy (SEM) of the *T. gondii* tachyzoites showing **a** normal, non-treated tachyzoites pointed anterior end and rounded posterior end and smooth regular surface (×20,000), **b** tachyzoite treated with compound **3d** showing multiple depressions and longitudinal deep ridges on its surface (arrow) (×20,000), **c** tachyzoite treated with compound **3d** showing leakage of cytoplasmic contents (arrow) (×20,000), **d** tachyzoite treated with compound **3e** showing a large membrane projection (arrow) (×20,000), **e** tachyzoite treated with compound **3e** showing sloughing of its surface (arrow) (×20,000), **f** tachyzoite treated with compound **3f** appear mutilated and disorganized with multiple projections (arrows) (×20,000), and **g** tachyzoite treated with compound **3f** showing multiple surface depressions and clefts (arrow) (×20,000)

To further explore the anti*-Toxoplasma* effect of the tested sulfa drug based 1,2,3-triazoles **3**(**d**,**e**,**f**), the ultrastructure of *T. gondii* tachyzoites treated with the three sulfa drugs was observed in comparison to normal, non-treated tachyzoites using scanning electron microscope (SEM). Typical, non-treated tachyzoites showed crescent-shaped parasite with pointed anterior end and normal posterior end and smooth regular surface (Fig. 2a). Tachyzoites treated with compound **3d** showed multiple depressions and longitudinal deep ridges with leakage of cytoplasmic contents (Fig. 2b and c). On the other hand, tachyzoites treated with compound **3e** showed large membrane projections and sloughing of its surface (Fig. 2d and e). Finally, those treated with compound **3f** showed the most evident morphological alterations, and disruption tachyzoites appear mutilated, disorganized with multiple projections and surface depressions and clefts (Fig. 2f and g).

### Transmission electron microscopic analysis

**Fig. 3:**
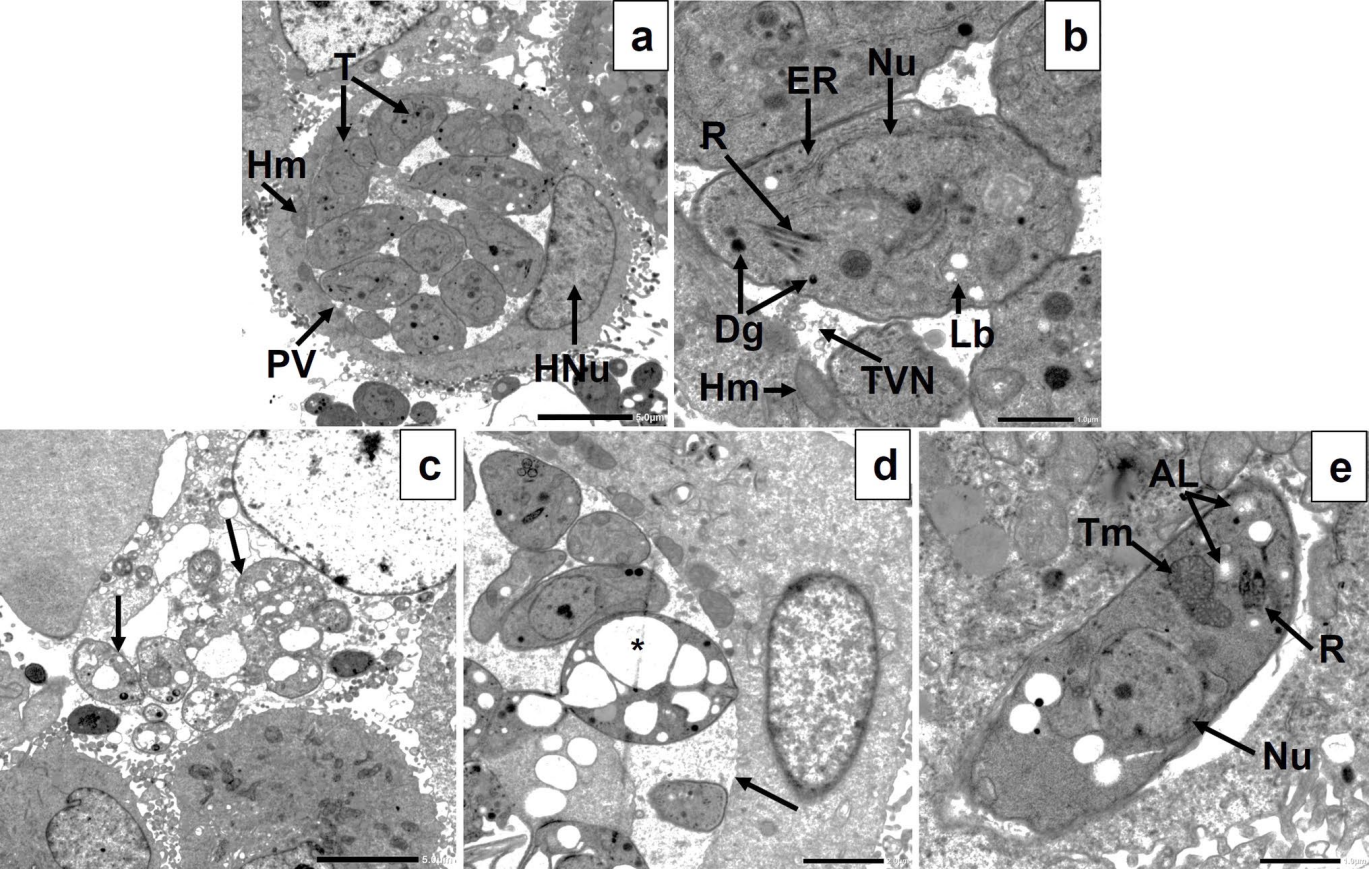
TEM images of *Toxoplasma gondii*-infected, non-treated (**a**, **b**) and compound **3d**-treated (**c**–**e**) Vero cells showing **a** section of infected, non-treated cell showing an intact host cell nucleus (HNu) and intracytoplasmic parasitophorous vacuole (PV) containing multiple tachyzoites (T) with the host cell mitochondria (Hm) adjacent to the cellular interface of the PV membrane (×1500); **b** longitudinal section of non-treated tachyzoite having intact plasma membrane and nucleus (Nu), endoplasmic reticulum (ER), rhoptries (R), dense granules (Dg), and lipid bodies (Lb). The host cell mitochondria (Hm) can also be seen. Normal tubulovesicular network (TVN) structure inside the PV surrounding the tachyzoites (×6000); **c** multiple extracellular compound **3d**-treated tachyzoites (arrows), released from a recently ruptured cell, containing multiple vacuoles (×1500); **d** multiple intracellular compound **3d**-treated tachyzoites within an intact PV membrane (arrow). Some of them appearing apparently normal, while others were vacuolated (asterisk) without nuclei or organelles (×3000); and **e** tachyzoite treated with compound **3d** showing amylopectin-like granules (AL) which disrupted apical complex and displaced the rhoptries (R). Apparently, normal nucleus (Nu) and tachyzoite mitochondria (Tm) could also be seen (×6000)

Transmission electron microscopic (TEM) examination was performed to identify the underlying mechanism of action of the three tested compounds on the intracellular tachyzoite over a longer period of treatment (24 h). The images of *T. gondii*-infected, non-treated cells showed an intact host cell nucleus and intracytoplasmic parasitophorous vacuole (PV) containing multiple tachyzoites together with the host cell mitochondria closely adjacent to the cellular interface of the PV membrane (Fig. 3a). The tachyzoites had intact plasma membranes, nuclei, endoplasmic reticulum, rhoptries, dense granules, and lipid bodies. The normal tubulovesicular network structure inside the PV surrounding the tachyzoites could also be detected (Fig. 3b).

**Fig. 4:**
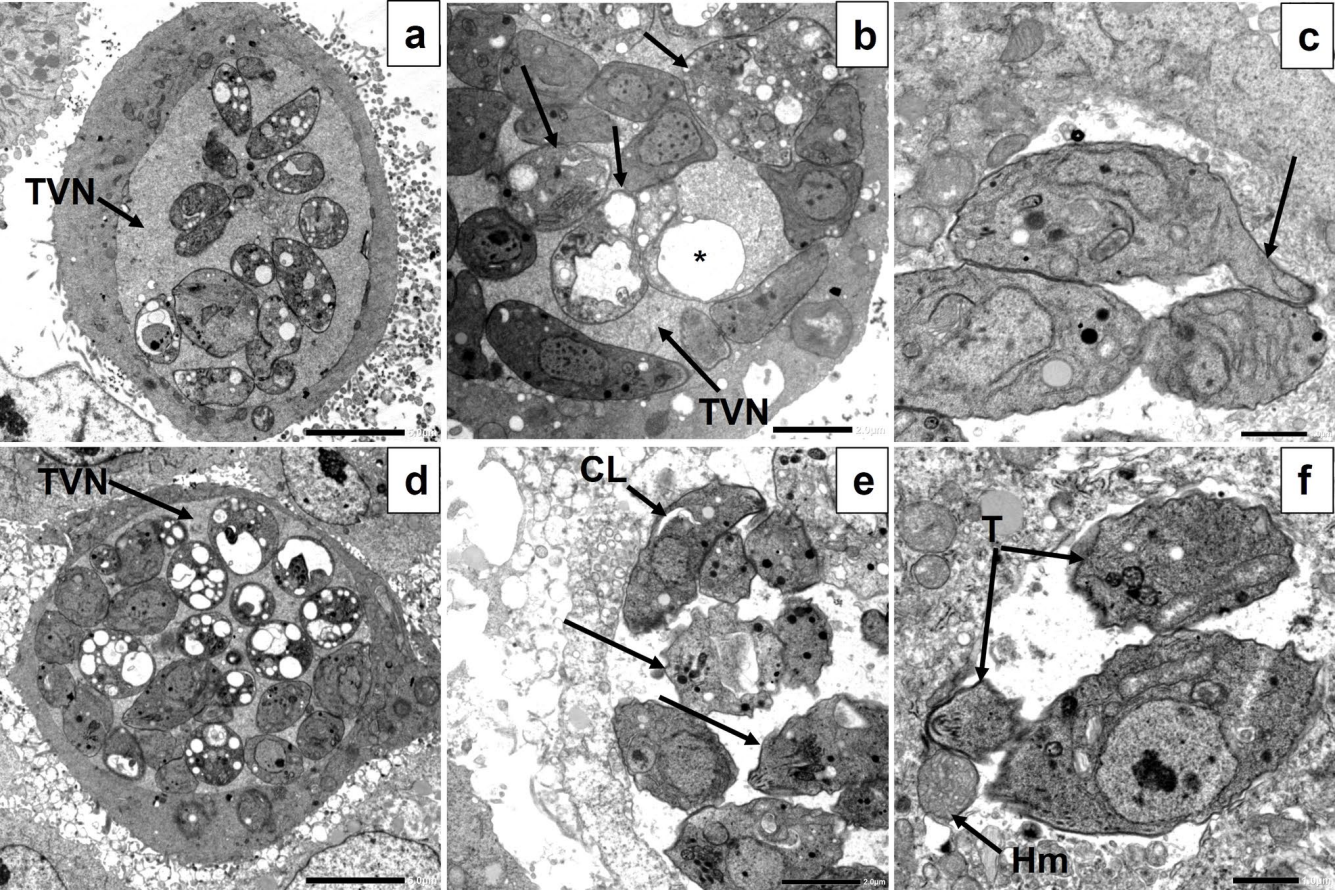
TEM images of *Toxoplasma gondii*-infected, compound **3e**-treated (**a**–**c**) and compound **3f**-treated (**d**–**e**) Vero cells showing **a** section of infected, compound **3e**-treated cell showing a large PV containing multiple vacuolated tachyzoites and abnormally dark and densely granular TVN (×1500); **b** multiple intracellular compound **3e**-treated tachyzoites within a PV. Some of them appear apparently normal, while others were vacuolated (arrows) without nuclei or organelles. The TVN appeared densely granular with large vacuoles (asterisk) (×3000); **c** tachyzoite treated with compound **3e** showing loss of cytoplasmic membrane integrity with appearance of elongated membrane projection (arrow) (×5000); **d** section of infected, compound **3f**-treated cell showing a large PV containing multiple vacuolated tachyzoites together with some apparently normal ones. TVN appeared dark and densely granular (×1500); **e** multiple intracellular compound **3f**-treated tachyzoites within a PV with some of them showing corrugated surface and intracytoplasmic vacuoles (arrows) while others had cytoplasmic clefts (CL) (×3000); and **f** at higher magnification, some tachyzoites appeared as if they were torn apart (T). Host cell mitochondria (Hm) could be seen inside the PV indicating loss of PV membrane integrity (×5000)

On the hand, intracellular tachyzoites treated with compound **3d** showed a range of morphological presentations as some of them appeared apparently normal, while others were vacuolated without nuclei or organelles (Fig. 3c, d). At higher magnification, they showed amylopectin-like granules which disrupted apical complex and displaced the rhoptries (Fig. 3e). Similarly, cells treated with compound **3e** showed large PVs containing multiple vacuolated tachyzoites without nuclei or organelles in addition to loss of cytoplasmic membrane integrity with appearance of elongated membrane projection, while others seemed apparently normal (Fig. 4a–c). Surprisingly, the tubulovesicular network appeared abnormally dark and densely granular with large vacuoles (Fig. 4a, b). Finally, cells treated with compound **3f** showed a similar appearance to those treated with compound **3e** with large PVs containing multiple vacuolated tachyzoites together with some apparently normal ones. The tubulovesicular network seemed also dark and densely granular (Fig. 4d). Yet, at higher magnification, some tachyzoites showed strangely corrugated surface while others had cytoplasmic clefts (Fig. 4e). Moreover, some tachyzoites appeared as if they were torn apart, and in addition, host cell mitochondria could be seen inside the PV indicating loss of PV membrane integrity (Fig. 4f).

## Discussion

Particularly in tropical areas, parasitic infections continue to have a significant negative influence on human health. Due to the lack of a viable anti*-Toxoplasma* vaccination and the ongoing danger of treatment resistance, the development of innovative anti-parasitic chemotherapies continues to be of crucial relevance for the management of toxoplasmosis (Said et al. 2021).

Numerous triazole analogs are still in clinical studies for the treatment of certain parasites; others have been licensed for use in hospitals and clinics (Said et al. 2021). The findings of the previous investigations renewed our interest in synthesizing such hybrid molecules. As a consequence, in the current investigation, anti*-Toxoplasma* drugs, that are highly active comparable to the positive control medication sulfadiazine, were produced by molecular hybridization of sulfonamide moieties and 1,2,3-triazole rings. A key therapeutic factor in the development of potent anti-parasitic activity of the targeted scaffolds might be the integration of the triazole skeletal moiety with the amino or hydroxyl group to the sulfa drugs **3**(**a**–**f**) (Viegas-Junior et al. 2007; Zhang et al. 2021). Thus, the results revealed that the synthesized sulfa drug derivatives **3**(**a**–**f**) had comparable lipophilicity and activities against *T. gondii* (Chen et al. 2018).

From the cytotoxicity results using **CC**_**50**_, it was found that the safety margin range of sulfadiazine (reference drug) was higher than the investigated sulfa drugs **3**(**a**–**f**). However, the **3**(**a**–**f**) showed more potent anti-*Toxoplasma* activity compared to sulfadiazine with much lower **IC**_**50**_ values with compound **3f** having tenfold lower **IC**_**50**_ **(0.01835 μM)** than sulfadiazine **(0.1852 μM)**. The present study revealed that the growth inhibition (**IC**_**50**_) of the tested compounds **3**(**a**–**f**) was high at very low concentrations compared to the standard reference sulfadiazine, while the safety of sulfadiazine was higher. Therefore, we used the selectivity index (SI*) to express the in vitro efficacy of a compound in the inhibition of *T. gondii* proliferation as the SI* is used to express the degree of anti-*Toxoplasma* activity where the higher SI* ratio, the more theoretically effective the compound (Hopper et al. 2019). The SI* ratio of **3**(**a**–**f**) exhibited values of **10.4**, **8.9**, **25.4**, **21**, **8.3**, **and 29**, respectively, which means that all of them are more effective than the positive control drug, which has a selectivity index of **6.9**.

Analogous to the current first-line therapy for toxoplasmosis, the tested compounds rely on inhibition of the folate pathway in the parasite (Anderson 2005; Wei et al. 2015). As previously mentioned, the sulfonamide component inhibits dihydropteroate synthase which in turn inhibits the parasite growth by blocking the biosynthesis of tetrahydrofolate, an essential factor needed for the production of nucleic acids which are required for DNA synthesis (Said et al. 2021). The obtained results were in accordance with the study reported the synthesis of series of non-peptide inhibitors bearing 1,2,3-triazole moieties toward the polo-box domain (PBD) of polo-like kinase reported by Chen et al*.*(Chen et al. 2018). Other molecular conjugates tethering triazole core were found to have a similar mechanism of action by blocking lipid biosynthesis (Kumar et al. 2014; Zhang et al. 2017).

The polar groups were anchored to the triazole ring to assess the influence of the pharmacological effect. The polar terminal groups, which were attached to the triazole ring, provide information on the characteristics of a chemical passive diffusion over a biological membrane through their H bonding capability with the receptors (Yamini and Vijjulatha 2008). Partition coefficient values between **3**(**a**–**f**) are usually targeted, while values between **3f** and **3c** are perfect where the derivative of high H-bonding ability had positive effect on their activity as anti-*Toxoplasma* compounds (Lipinski et al. 2012), (Tables 1 and 2). It gives a clear understanding about the transport characteristics of a chemical across a biological membrane through passive diffusion (Saeedi et al. 2019). Furthermore, the three hybrid configurations of the tested offshoots **3**(**d**–**f**) contain the most effective polar group (NH_2_) which is more interactive with the receptor protein via hydrogen bonding resulting in a great increase of their biological activity (Craik et al. 2013). Therefore, the three synthetic products **3**(**d**–**f**) can be considered as potential future therapeutic agents for toxoplasmosis. The most effective polar group (NH_2_) increased biological features are due to its hydrogen-bonding capacity, mild dipole character, stiffness, and stability under in vivo settings (Zhang et al. 2017).

To study the impact of the studied derivatives **3**(**d**–**f**) on the surface of *T. gondii* at high magnification, scanning electron microscopy (SEM) was used (El-Tombary et al. 1999). All of these compounds caused significant morphological changes in the extracellular parasites over a period of 2 h of exposure. Tachyzoites treated with compound **3f** showed the most drastic morphologic alterations in the form of parasite mutilation. These changes may indicate the inability of the organism to enter into the host cells leading to their elimination (Aikawa et al. 1977; Hammouda et al. 1992). In addition, the tachyzoites treated with derivative **3d** showed some changes but to lesser extent than **3f** with surface irregularities and leakage of cytoplasmic contents. Those treated with compound **3e** showed similar morphological changes in the form of surface sloughing and large membrane projections. All of these changes caused by compounds **3**(**d**–**f**) lead to changes in the shape of the organism which could be attributed to interference of the drugs with DNA synthesis of the parasite as a result of interfering with folic acid synthesis (Chulay et al. 1984).

The observations obtained by SEM were validated by TEM analysis, which also assisted in identifying potential underlying mechanisms of action of the three experienced drugs **3**(**d**–**f**) on the intracellular tachyzoites over a longer duration of treatment. All three compounds significantly altered the ultrastructure, causing cytoplasmic vacuolations to emerge, parasite organelles to vanish, and apical complex structures to be disrupted (Portes et al. 2018). Moreover, loss of cytoplasmic membrane integrity with appearance of membrane projections and extensions was observed especially in compound **3d** and **3e**. The most dramatic alteration was observed with compound **3f** with cytoplasmic clefts and even completely torn apart parasite. These drastic cellular disruptions could indicate cell death by apoptosis as it has been previously reported that tachyzoites cytoplasmic vacuolation and clefts are markers of apoptotic cell death (Said et al. 2021). Furthermore, appearance of cytoplasmic structures resembling amylopectin following treatment with compound **3e**. Amylopectin granules could be detected in the bradyzoite stage as part of the resistance mechanism to stress conditions in the cellular microenvironment, which includes nutrient shortage, temperature variations, pH fluctuations, and IFNγ-induced immune response (Djurković-Djaković et al. 2005; Eaton et al. 2006). This might indicate that this particular compound induced parasite stress which might have triggered stage conversion to bradyzoites. This is an adaptive response of the parasite toward the treatment, because bradyzoites are less sensitive to treatment due to their lower metabolic rate (Eng et al. 1991). The tubulovesicular network seemed also dark and densely granular after treatment with compounds **3e** and **3f**; however, loss of PV membrane integrity was only noted after treatment with compound **3f**. Normally, the host cell supplies nutrition to the parasite through the formation of a network of tubules and vesicles (the tubulovesicular network). This disintegrated granular appearance after treatment could be explained by the leakage of the cytoplasmic contents through the disrupted cytoplasmic membrane of affected tachyzoites (Elkerdany et al. 2020). Thus, it can be assumed that treatment compromised the integrity of this essential network and its surrounding membrane.

Recently, Almeida-Souza et al. (Almeida-Souza et al. 2020) showed the in vitro anti-leishmanial activity of some 1,4-disubstituted-1,2,3-triazole compounds and their effect on the ultrastructure of the promastigote form of *Leishmania amazonensis* and described the drug-likeness and pharmacokinetic which changed the nuclear chromatin, nuclear membrane, trace of kinetoplast with electron-density loss, and loss in cytoplasm organelles (nucleus, kinetoplast, mitochondria), supporting our results and confirming that the synthesized 1,2,3-triazole-sulfonamide molecular conjugates **3**(**d**–**f**) are promising drugs to eliminate the parasites. The low dose from these compounds obtained high protection and reduced adverse side effects and toxicity. The observed effects of these tested candidates may offer direction for future in vivo studies and clinical trials for their anti*-Toxoplasma* potency.

Additionally, it has been reported that treatment with sulfadiazine alone is characterized by a cytostatic effect where there is a decline in the number of intracellular parasites, but without significant morphological changes to the parasite (Derouin and Chastang 1989). Portes et al. (Portes et al. 2018) found no significant ultrastructural alterations in tachyzoites after treatment with sulfadiazine as the only noticed morphological change was the appearance of amylopectin-like granules, which is an ultrastructural sign of bradyzoites, suggesting that sulfadiazine induced stage conversion, verifying its cytostatic effect. On the contrary, hybrids of 1,2,3-triazole and sulfonamide, evaluated in the current study, were more effective than sulfadiazine alone, as parasite growth reduction was accompanied by damaged and altered morphology, indicating a cytopathic effect. This suggests that these hybrids of 1,2,3-triazole and sulfonamide may inhibit *T. gondii* by inducing apoptotic cell death. All of the changes observed after treatment should be further investigated to learn more about cell death induced in the parasites.

## Conclusion

Treatment failures, observed with already existing drugs, have been linked to host characteristics such as drug intolerance and malabsorption as well as the emergence of drug resistance among parasites. Furthermore, the currently approved drugs are not well tolerated by patients leading to serious and life-threatening adverse reactions necessitating treatment cessation in some cases. High doses required in treatment may be toxic to the patient. Thus, we focused our design on imitating the approved medications, particularly the most potent amino groups, which are known to be important in the interaction with the receptor protein via hydrogen bonding, resulting in increased biological activity. For the purpose of assessing their inhibitory impact on *T. gondii* at a safe concentration, all experimental tests were conducted in vitro. All six sulfonamide-1,2,3-triazole molecular hybrids exhibited more activity than the commonly used drug sulfadiazine. The three new compounds of the NH_2_ derivatives **3**(**d**–**f**), displayed a potent activity compared to their OH analogs, **3**(**a**–**c**), and control sulfadiazine. Although, the target protein and mechanism of action of sulfa drugs on *T. gondii* is still unclear, the inhibitory effect of our designed derivatives **3**(**d**–**f**) has been attributed to blocking the biosynthesis of tetrahydrofolate, an essential factor needed to produce nucleic acids which are required for DNA synthesis as well as the destruction of cell membranes of *T. gondii* by blocking lipid biosynthesis. In addition, the low IC_50_ of these novel-targeted 1,2,3-triazole-sulfonamide molecular conjugates, that mimic and even surpass the licensed medications’ precisely matched inhibitory capabilities of *T. gondii* at lower concentrations, could offer a tremendous opportunity in the field of *Toxoplasma* research*.* The observed effects of these interesting sulfa drugs bearing triazole rings may usher in the right direction for future in vivo research and clinical trials.

## Supplementary information

ESM 1

## Data Availability

The data presented during the current study are available from the corresponding author on request.

## References

[CR1] Aikawa M, Komata Y, Asai T, Midorikawa O (1977). Transmission and scanning electron microscopy of host cell entry by Toxoplasma gondii. Am J Pathol.

[CR2] Aljohani FS (2022). Synthesis, characterization and nanoformulation of novel sulfonamide-1, 2, 3-triazole molecular conjugates as potent antiparasitic agents. Int J Mol Sci.

[CR3] Al-Malki ES (2021). Toxoplasmosis: stages of the protozoan life cycle and risk assessment in humans and animals for an enhanced awareness and an improved socio-economic status. Saudi J Biol Sci.

[CR4] Almeida-Souza F (2020). 1, 4-Disubstituted-1, 2, 3-Triazole compounds induce ultrastructural alterations in leishmania amazonensis promastigote: an in vitro antileishmanial and in silico pharmacokinetic study. Int J Mol Sci.

[CR5] Anderson AC (2005). Targeting DHFR in parasitic protozoa. Drug Discov Today.

[CR6] Bérubé G (2016). An overview of molecular hybrids in drug discovery. Expert Opin Drug Discovery.

[CR7] Boyle JP, Saeij JP, Coller SP, Boothroyd JC (2006). Polymorphic secreted kinases are key virulence factors in toxoplasmosis. Am J Trop Med Hyg.

[CR8] Carvalho C, De Melo E (2010). Anti-parasitic action and elimination of intracellular Toxoplasma gondii in the presence of novel thiosemicarbazone and its 4-thiazolidinone derivatives. Braz J Med Biol Res.

[CR9] Celik F, Unver Y, Barut B, Ozel A, Sancak K (2018). Synthesis, characterization and biological activities of new symmetric bis-1, 2, 3-triazoles with click chemistry. Med Chem.

[CR10] Chen Y (2018). Identification of novel and selective non-peptide inhibitors targeting the polo-box domain of polo-like kinase 1. Bioorg Chem.

[CR11] Choi W-Y (1997). Foodborne outbreaks of human toxoplasmosis. J Infect Dis.

[CR12] Chou T-C (2006). Theoretical basis, experimental design, and computerized simulation of synergism and antagonism in drug combination studies. Pharmacol Rev.

[CR13] Chou T-C, Talaly P (1977). A simple generalized equation for the analysis of multiple inhibitions of Michaelis-Menten kinetic systems. J Biol Chem.

[CR14] Chulay JD, Watkins WM, Sixsmith DG (1984). Synergistic antimalarial activity of pyrimethamine and sulfadoxine against Plasmodium falciparum in vitro. Am J Trop Med Hyg.

[CR15] Conseil V, Soete M, Dubremetz J (1999). Serine protease inhibitors block invasion of host cells by Toxoplasma gondii. Antimicrob Agents Chemother.

[CR16] Craik DJ, Fairlie DP, Liras S, Price D (2013). The future of peptide-based drugs. Chem Biol Drug Des.

[CR17] de Souza W, Attias M (2018). New advances in scanning microscopy and its application to study parasitic protozoa. Exp Parasitol.

[CR18] Derouin F, Chastang C (1989). In vitro effects of folate inhibitors on Toxoplasma gondii. Antimicrob Agents Chemother.

[CR19] Diab M, El-Bahy M (2008). Toxoplasma gondii: virulence of tachyzoites in serum free media at different temperatures. Exp Parasitol.

[CR20] Djurković-Djaković O, Nikolić A, Bobić B, Klun I, Aleksić A (2005). Stage conversion of Toxoplasma gondii RH parasites in mice by treatment with atovaquone and pyrrolidine dithiocarbamate. Microbes Infect.

[CR21] Dubey JP (2016). Toxoplasmosis of animals and humans.

[CR22] Eaton MS, Weiss LM, Kim K (2006). Cyclic nucleotide kinases and tachyzoite–bradyzoite transition in Toxoplasma gondii. Int J Parasitol.

[CR23] Elkerdany ED, Elnassery SM, Arafa FM, Zaki SA, Mady RF (2020). In vitro effect of a novel protease inhibitor cocktail on Toxoplasma gondii tachyzoites. Exp Parasitol.

[CR24] El-Tombary AA, Ismail KA, Aboulwafa OM, Omar A-MM, El-Azzouni MZ, El-Mansoury ST (1999). Novel triazolo [4, 3-a] quinazolinone and bis-triazolo [4, 3-a: 4, 3′-c] quinazolines: synthesis and antitoxoplasmosis effect. Il Farmaco.

[CR25] Eng R, Padberg F, Smith S, Tan E, Cherubin C (1991). Bactericidal effects of antibiotics on slowly growing and nongrowing bacteria. Antimicrob Agents Chemother.

[CR26] Ertl P, Rohde B, Selzer P (2000). Fast calculation of molecular polar surface area as a sum of fragment-based contributions and its application to the prediction of drug transport properties. J Med Chem.

[CR27] Galal L, Hamidović A, Dardé ML, Mercier M (2019). Diversity of Toxoplasma gondii strains at the global level and its determinants. Food Waterborne Parasitol.

[CR28] Guo H, Gao Y, N'Da DD, Xuan X (2021). In vitro anti-Toxoplasma gondii efficacy of synthesised benzyltriazole derivatives. Onderstepoort J Vet Res.

[CR29] Hammouda N, El-Mansoury S, El-Azzouni M (1992). Toxoplasma gondii: scanning electron microscopic study before and after treatment. J Trop Med.

[CR30] Hermes G (2008). Neurological and behavioral abnormalities, ventricular dilatation, altered cellular functions, inflammation, and neuronal injury in brains of mice due to common, persistent, parasitic infection. J Neuroinflammation.

[CR31] Hernandez AV (2017). A systematic review and meta-analysis of the relative efficacy and safety of treatment regimens for HIV-associated cerebral toxoplasmosis: is trimethoprim-sulfamethoxazole a real option?. HIV Med.

[CR32] Hopper AT (2019). Discovery of selective Toxoplasma gondii dihydrofolate reductase inhibitors for the treatment of toxoplasmosis. J Med Chem.

[CR33] Howe DK, Sibley LD (1995). Toxoplasma gondii comprises three clonal lineages: correlation of parasite genotype with human disease. J Infect Dis.

[CR34] Huisgen R (1963). 1, 3-dipolar cycloadditions. Past and future. Angew Chem Int Ed Engl.

[CR35] Jeliński T, Przybyłek M, Cysewski P (2019). Solubility advantage of sulfanilamide and sulfacetamide in natural deep eutectic systems: experimental and theoretical investigations. Drug Dev Ind Pharm.

[CR36] Khosravi M, Mohammad Rahimi H, Doroud D, Mirsamadi ES, Mirjalali H, Zali MR (2020). In vitro evaluation of mannosylated paromomycin-loaded solid lipid nanoparticles on acute toxoplasmosis. Front Cell Infect Microbiol.

[CR37] Kongsaengdao S, Samintarapanya K, Oranratnachai K, Prapakarn W, Apichartpiyakul C (2008). Randomized controlled trial of pyrimethamine plus sulfadiazine versus trimethoprim plus sulfamethoxazole for treatment of toxoplasmic encephalitis in AIDS patients. J Int Assoc Physicians AIDS Care.

[CR38] Kumar S, Khokra SL, Yadav A (2021). Triazole analogues as potential pharmacological agents: a brief review. Future J Pharm Sci.

[CR39] Kumar S, Prahalathan P, Saravanakumar M, Raja B (2014). Vanillic acid prevents the deregulation of lipid metabolism, endothelin 1 and up regulation of endothelial nitric oxide synthase in nitric oxide deficient hypertensive rats. Eur J Pharmacol.

[CR40] Lipinski CA, Lombardo F, Dominy BW, Feeney PJ (2012). Experimental and computational approaches to estimate solubility and permeability in drug discovery and development settings. Adv Drug Deliv Rev.

[CR41] McGettigan BD, Hew M, Phillips E, McLean-Tooke A (2012). Sulphadiazine-induced renal stones in a 63-year-old HIV-infected man treated for toxoplasmosis. Case Rep Dermatol.

[CR42] Molina DA (2021). In vitro evaluation of new 4-thiazolidinones on invasion and growth of Toxoplasma gondii. Int J Parasitol Drugs Drug Resist.

[CR43] Montazeri M (2020). Anti-Toxoplasma activities of the hydroalcoholic extract of some brassicaceae species. Adv Biomed Res.

[CR44] Ozgonul C, Besirli CG (2017). Recent developments in the diagnosis and treatment of ocular toxoplasmosis. Ophthalmic Res.

[CR45] Paredes-Santos T, Martins-Duarte E, Vitor R, De Souza W, Attias M, Vommaro R (2013). Spontaneous cystogenesis in vitro of a Brazilian strain of Toxoplasma gondii. Parasitol Int.

[CR46] Park Y-H, Han J-H, Nam H-W (2011). Clinical features of ocular toxoplasmosis in Korean patients. Korean J Parasitol.

[CR47] Pink R, Hudson A, Mouriès M-A, Bendig M (2005). Opportunities and challenges in antiparasitic drug discovery. Nat Rev Drug Discov.

[CR48] Portes JA (2018). A new iron (III) complex-containing sulfadiazine inhibits the proliferation and induces cystogenesis of Toxoplasma gondii. Parasitol Res.

[CR49] Ryu B-Y, Emrick T (2011). Bisphenol-1, 2, 3-triazole (BPT) epoxies and cyanate esters: synthesis and self-catalyzed curing. Macromolecules.

[CR50] Saeedi M (2019). Design and synthesis of novel quinazolinone-1, 2, 3-triazole hybrids as new anti-diabetic agents: in vitro α-glucosidase inhibition, kinetic, and docking study. Bioorg Chem.

[CR51] Sahu A, Sahu P, Agrawal R (2020). A recent review on drug modification using 1, 2, 3-triazole. Curr Chem Biol.

[CR52] Said MA (2021). New 1, 2, 3-triazole scaffold schiff bases as potential anti-COVID-19: design, synthesis, DFT-molecular docking, and cytotoxicity aspects. Vaccines.

[CR53] Sanchez SG, Besteiro S (2021). The pathogenicity and virulence of Toxoplasma gondii. Virulence.

[CR54] Saraf P, Shwab EK, Dubey JP, Su C (2017). On the determination of Toxoplasma gondii virulence in mice. Exp Parasitol.

[CR55] Shaw MK, Roos DS, Tilney LG (2002). Cysteine and serine protease inhibitors block intracellular development and disrupt the secretory pathway of Toxoplasma gondii. Microbes Infect.

[CR56] Smith CL, Powell KR (2000). Review of the sulfonamides and trimethoprim. Pediatr Rev.

[CR57] Thebault A, Kooh P, Cadavez V, Gonzales-Barron U, Villena I (2021). Risk factors for sporadic toxoplasmosis: a systematic review and meta-analysis. Microbial Risk Analysis.

[CR58] Viegas-Junior C, Danuello A, da Silva BV, Barreiro EJ, Fraga CAM (2007). Molecular hybridization: a useful tool in the design of new drug prototypes. Curr Med Chem.

[CR59] Wei H-X, Wei S-S, Lindsay DS, Peng H-J (2015). A systematic review and meta-analysis of the efficacy of anti-Toxoplasma gondii medicines in humans. PloS One.

[CR60] Winey M, Meehl JB, O'Toole ET, Giddings TH (2014). Conventional transmission electron microscopy. Mol Biol Cell.

[CR61] Wong S-Y, Remington JS (1993). Biology of Toxoplasma gondii. AIDS (London, England).

[CR62] Yamini L, Vijjulatha M (2008). Inhibitors of human dihydrofolate reductase: a computational design and docking studies using glide. E-Journal of Chemistry.

[CR63] Zhang R-H, Jin R, Deng H, Shen Q-K, Quan Z-S, Jin C-M (2021). Evaluation of the anti-Toxoplasma gondii activity of hederagenin in vitro and in vivo. Korean J Parasitol.

[CR64] Zhang S (2017). Triazole derivatives and their anti-tubercular activity. Eur J Med Chem.

