# Supplementary material for: Sulfadiazine analogs: anti-Toxoplasma in vitro study of sulfonamide triazoles
Source: Parasitol Res. 2023 Aug 23;122(10):2353–65. doi: 10.1007/s00436-023-07936-x (PMC10495491; doi:10.1007/s00436-023-07936-x)
Supplement: Supplementary file 1 — ESM 1 [file 436_2023_7936_MOESM1_ESM.docx]

**Supplementary Data**

# **Sulfadiazine analogues: anti*-*toxoplasma *in vitro* study of *s*ulfonamide triazoles**

**Fadwa M Arafa^1*^, Doaa Hassan Osman^2^, Mona Mohamed Tolba^2^, Nadjet Rezki^3^, Mohamed R Aouad^3^, Mohamed Hagar^4*^, Mervat Osman^2^, Heba Said^2^**

1. Department of Medical Parasitology, Faculty of Medicine, Alexandria University, Alexandria 21577, Egypt
2. Department of Parasitology Medical Research Institute Alexandria University, Alexandria 21561, Egypt
3. Department of Chemistry, College of Science, Taibah University, Al-Madinah Al-Munawarah 30002, Saudi Arabia
4. Department of Chemistry, Faculty of Science, Alexandria University, Alexandria 21321, Egypt

* Correspondence: f_arafa10@alexmed.edu.eg (F.M.A.)

***4-(4-(Aminomethyl)-1H-1,2,3-triazol-1-yl)-N-(pyrimidin-2-yl)benzenesulfonamide (3a)****.* It was obtained in 86 % yield as yellow powder; Mp: 220-221 °C. IR (KBr) ύ_max_/cm^-1^: 3380-3450 (NH, NH_2_), 3045 (CH_a5_), 2940 (CH_al_), 1620 (C=N), 1560 (C=C). ^1^H-NMR (DMSO-*d*_6_, 400 MHz): δ_H_ = 4.25 (s, 2H, C**H_2_**), 6.84 (s, 2H, N**H_2_**), 7.04 (bs, 1H, Ar-**H**), 8.16-8.43 (m, 6H, Ar-**H**), 8.50 (s, 1H, **H**-5-triazolyl), 12.02 (s, 1H, N**H**SO_2_). ^13^C-NMR (DMSO-*d*_6_, 100 MHz): δ_C_ = 56.56 (**C**H_2_); 119.97, 120.12, 121.44, 122.75, 127.85, 128.33, 139.25, 146.36, 150.23, 158.64, 159.25 (Ar-**C, C**=N). Calculated for C_13_H_13_N_7_O_2_S: C: 47.12; H; 3.95; N; 29.59. Found: C: 47.29; H, 3.78; N, 29.34. HRMS (ESI): 331.0654 [M^+^].

***4-(4-(Aminomethyl)-1H-1,2,3-triazol-1-yl)-N-(pyridin-2-yl)benzenesulfonamide (3b)***. It was obtained in 84 % yield as yellow solid; Mp: 248-250 °C. IR (KBr) ύ_max_/cm^-1^: 3350-3440 (NH, NH_2_), 3060 (CH_ar_), 2920 (CH_al_), 1610 (C=N), 1550 (C=C). ^1^H-NMR (DMSO-*d*_6_, 400 MHz): δ_H_ = 4.42 (s, 2H, C**H_2_**), 6.85 (s, 1H, N**H_2_**), 7.23-7.51 (m, 2H, Ar-**H**), 7.76-8.05 (m, 4H, Ph-**H**), 8.39 (bs, 2H, Ar-**H**), 8.88 (s, 1H, **H**-5-triazolyl), 12.43 (s, 1H, N**H**SO_2_). ^13^C-NMR (DMSO-*d*_6_, 100 MHz): δ_C_ = 56.49 (**C**H_2_); 119.73, 121.22, 122.11, 123.87, 128.22, 129.45, 140.45, 148.76, 154.11, 158.07, 159.75 (Ar-**C, C**=N). Calculated for C_14_H_14_N_6_O_2_S: C: 50.90; H: 4.27; N: 25.44. Found: C: 50.78; H: 4.42; N: 25.67. HRMS (ESI): 330.0689 [M^+^].

***N-(Diaminomethylene)-4-(4-(aminomethyl)-1H-1,2,3-triazol-1-yl)benzene-sulfonamide (3c)***. It was obtained in 88 % yield as yellow pale powder; Mp: 278-279 °C. IR (KBr) ύ_max_/cm^-1^: 3310-3460 (NH_2_), 3080 (CH_ar_), 2960 (CH_al_), 1615 (C=N), 1580 (C=C). ^1^H-NMR (DMSO-*d*_6_, 400 MHz): δ_H_ = 4.43 (s, 2H, C**H_2_**), 6.78 (bs, 6H, 3 x N**H_2_**), 7.94-8.07 (m, 4H, Ar-**H**), 8.72 (s, 1H, **H**-5-triazolyl). ^13^C-NMR (DMSO-*d*_6_, 100 MHz): δ_C_ = 57.09 (**C**H_2_); 118.87, 121.09, 121.22, 124.87, 126.66, 128.23, 138.39, 147.45, 156.26, 155.45, 157.29, 158.45 (Ar-**C**, **C**=N). Calculated for C_10_H_13_N_7_O_2_S: C: 40.67; H: 4.44; N: 33.20. Found: C: 40.49; H: 4.67; N: 33.35. HRMS (ESI): 295.0599 [M^+^].

***Characterization of 4-(4-(hydroxymethyl)-1H-1,2,3-triazol-1-yl)-N-(pyrimidin-2-yl)benzenesulfonamide (3d).*** It was obtained as white crystal in 88 % yield; Mp: 185-186 °C. IR (KBr) ύ_max_/cm^-1^: 1582 (C=C), 1639 (C=N), 2899, 2947 (Al.C-H), 3b6 (Ar.C-H), 33c(NH), 3487 cm^-1^ (OH). ^1^H NMR (DMSO-*d*_6_, 400 MHz):δ_H_ = 12.10 (1H, s, N**H**SO_2_), 8.79 (1H, s, C**H**-1,2,3-triazole), 8.53 (2H, bs, Ar-**H**), 8.15 (4H, bs, Ph-**H**), 7.07 (1H, bs, Ar-**H**), 5.41 (1H, s, O**H**), 4.61 (2H, s, C**H_2_**NH_2_). ^13^C NMR (DMSO-*d*_6_, 100 MHz):δ_C_ = 158.95, 158.85, 149.93, 144.56, 138.75, 127.99, 127.78, 121.65, 121.58, 120.42, 120.27 (**C**=N, Ar-**C**), 55.16 (**C**H_2_). Calculated for C_13_H_12_N_6_O_3_S: C: 46.98; H: 3.64; N: 25.29. Found: C: 46.69; H: 3.35; N: 25.08. HRMS (ESI): 332.0443 [M^+^].

***Characterization of 4-(4-(hydroxymethyl)-1H-1,2,3-triazol-1-yl)-N-(pyridin-2-yl)benzenesulfonamide(3e).***

It was obtained as yellow crystal in 86 % yield; Mp: 223-224 °C. IR (KBr) ύ_max_/cm^-1^: 1577 (C=C), 1642 (C=N), 2889, 2956 (Al.C-H), 3082 (Ar.C-H), 3323(NH), 3498 cm^-1^ (OH). ^1^H NMR (DMSO-*d*_6_, 400 MHz):δ_H_ = 12.58 (1H, s, N**H**SO_2_), 8.80 (1H, s, C**H**-1,2,3-triazole), 8.13 (4H, bs, Ph-**H**), 6.87-7.20 (4H, m, Ar-**H**), 5.38 (1H, s, O**H**), 4.62 (2H, s, C**H_2_**NH_2_). ^13^C NMR (DMSO-*d*_6_, 100 MHz):δ_C_ = 169.90, 163.73, 158.90, 157.86, 149.82, 147.99, 139.26, 132.93, 129.40, 128.47, 121.21, 120.12, 119.99 (**C**=N, Ar-**C**), 55.31 (**C**H_2_). Calculated for C_14_H_13_N_5_O_3_S: C: 50.75; H: 3.95; N: 21.14. Found: C: 50.38; H: 3.55; N: 21.47. HRMS (ESI): 331.0598 [M^+^].

***Characterization of N-(diaminomethylene)-4-(4-(hydroxymethyl)-1H-1,2,3-triazol-1-yl)benzenesulfonamide (3f).*** It was obtained as yellow pale crystal in 90 % yield; Mp: 254-256 °C. IR (KBr) ύ_max_/cm^-1^: 1577 (C=C), 1642 (C=N), 2889, 2956 (Al.C-H), 3082 (Ar.C-H), 3319-3376 (NH_2_), 3509 cm^-1^ (OH). ^1^H NMR (DMSO-*d*_6_, 400 MHz):δ_H_ = 8.78 (1H, s, C**H**-1,2,3-triazole), 8.05 (2H, d, *J* = 4Hz, Ar-**H**), 7.93 (2H, d, *J* = 4Hz, Ar-H), 6.90 (4H, s, 2xN**H_2_**), 5.45 (1H, s, O**H**), 4.62 (2H, s, C**H_2_**). ^13^C NMR (DMSO-*d*_6_, 100 MHz):δ_C_ = 158.31, 149.42, 144.22, 144.02, 138.58, 127.75, 121.87, 120.59, 119.99 (**C**=N, Ar-**C**), 55.54 (**C**H_2_). Calculated for C_10_H_12_N_6_O_3_S: C: 40.54; H: 4.08; N: 28.36. Found: C: 40.89; H: 4.37; N: 28.59. HRMS (ESI): 296.0338 [M^+^].
